# Effect of reproductive factors on stage, grade and hormone receptor status in early-onset breast cancer

**DOI:** 10.1186/bcr1198

**Published:** 2005-05-16

**Authors:** Joan A Largent, Argyrios Ziogas, Hoda Anton-Culver

**Affiliations:** 1 Epidemiology Division, Department of Medicine, University of California, Irvine, Irvine, California, USA

## Abstract

**Introduction:**

Women younger than 35 years who are diagnosed with breast cancer tend to have more advanced stage tumors and poorer prognoses than do older women. Pregnancy is associated with elevated exposure to estrogen, which may influence the progression of breast cancer in young women. The objective of the present study was to examine the relationship between reproductive events and tumor stage, grade, estrogen receptor and progesterone receptor status, and survival in women diagnosed with early-onset breast cancer.

**Methods:**

In a population-based, case–case study of 254 women diagnosed with invasive breast cancer at age under 35 years, odds ratios (ORs) and 95% confidence intervals (CIs) were estimated using unconditional logistic regression with tumor characteristics as dependent variables and adjusting for age and education. Survival analyses also examined the relationship between reproductive events and overall survival.

**Results:**

Compared with nulliparous women, women with three or more childbirths were more likely to be diagnosed with nonlocalized tumors (OR = 3.1, 95% CI = 1.3–7.7), and early age (<20 years) at first full-term pregnancy was also associated with a diagnosis of breast cancer that was nonlocalized (OR = 3.0, 95% CI = 1.2–7.4) and of higher grade (OR = 3.2, 95% CI 1.0–9.9). The hazard ratio for death among women with two or more full-term pregnancies, as compared with those with one full-term pregnancy or none, was 2.1 (95% CI = 1.0–4.5), adjusting for stage. Among parous women, those who lactated were at decreased risk for both estrogen receptor and progesterone receptor negative tumors (OR = 0.2, 95% CI = 0.1–0.5, and OR = 0.4, 95% CI = 0.2–0.8, respectively).

**Conclusion:**

The results of the present study suggest that pregnancy and lactation may influence tumor presentation and survival in women with early-onset breast cancer.

## Introduction

Invasive breast cancer among women younger than 35 years is relatively rare in comparison with breast cancer in older women; only 2% of diagnoses occur in the younger age group [1]. However, it is the leading type of cancer and cause of cancer-related death in women aged 15–34 years [2]. Prognosis following diagnosis for younger women is worse; they have shorter recurrence-free and overall survival than do older premenopausal [3] and post-menopausal [4] women.

Malignant breast tumors in women younger than 35 years tend to have features that are characteristic of more aggressive or advanced tumors compared with tumors in older women. The tumors in younger women tend to be larger, have more nodal involvement, are more poorly differentiated, and are more often estrogen receptor (ER) negative than are tumors in older women [3,5]. These tumor variables have been associated with poorer overall survival. Although there is evidence linking tumor factors with recurrence-free and overall survival, little is known about host characteristics that may contribute to differences in tumor variables observed at diagnosis, particularly in young women, who as a group are not subject to the same screening practices (namely mammography) as their older counterparts. An understanding of host variables that influence stage at presentation and other tumor characteristics may help to elucidate the mechanisms that are involved in tumor progression in early-onset breast cancer.

Pregnancy and childbirth are generally believed to reduce a woman's long-term risk for breast cancer. However, some studies have documented a transient increase in risk in the years immediately following childbirth [6-8], and a decrease in survival for women diagnosed in the years following childbirth [9,10].

Early detection is a key factor in determining whether a woman will survive a breast cancer diagnosis. Advances in screening methods have lead to a significant decrease in the percentage of diagnoses of late-stage disease in the past decade [11]. However, women younger than 40 years are generally not included in recommendations regarding who should undergo screening mammograms in the USA.

The observation that women with breast cancer under age 35 years are more likely to be diagnosed with advanced stage disease [3,5] and to have poorer survival than older women, combined with the fact that these women are not included in mammogram screening recommendations, underscores the importance of uncovering the processes of disease progression in these women so that effective, targeted strategies for early detection and management of this disease may be developed. The present study examines the relationships between reproductive and hormonal factors, stage, grade, hormone receptor status, and survival among women diagnosed with breast cancer at age under 35 years.

## Materials and methods

### Study design

A population-based, case–case design was used to examine the relationships between host reproductive and other variables, and tumor characteristics at presentation in breast cancer patients diagnosed at an early age.

### Population under study

The study included 298 patients with early-onset (diagnosed at age <35 years) breast cancer. Eligible participants were all women aged between 18 and 34 years residing in one of 38 of the 58 California counties (*n *= 169), in Connecticut (*n *= 60), or in Massachusetts (*n *= 69) at the time of diagnosis. The participation rate was 65% for patients from California, 61% for patients from Connecticut, and 55% for patients from Massachusetts.

The women were diagnosed with invasive breast cancer between 1 January 1995 and 31 December 1996. The patients had to be living at the time of contact. Women who met these criteria were identified through the state or regional cancer registries of California, Connecticut, and Massachusetts. Twenty-eight women in California, one woman in Massachusetts, and no women in Connecticut died before study contact. Limited data on nonparticipants from California allowed for a comparison of demographic variables between participants and nonparticipants. Although there were no significant age differences between participants (mean 31.3 years) and nonparticipants (mean 30.9 years), participants (73%) were more likely to be non-Hispanic white than were nonparticipants (59%).

### Protocol

Before contact with any potential participants, approval was obtained from the local institutional review boards at participating centers. Consent to participate was first obtained from the treating physician. Potential participants were then mailed introductory letters and response forms. Trained interviewers telephoned participants to conduct a structured telephone interview and to schedule an appointment to collect a blood sample. Participants completed a written informed consent and a release of medical information form. The mean time between diagnosis and interview was 11.5 months (range 0–27 months).

### Data collection and measurement tools

The interviews obtained information on host characteristics including age, demographic information, reproductive variables (age at menarche, pregnancy and childbirth history, history of spontaneous or induced abortions, lactation history), self-reported family history of cancer, and oral contraceptive and other hormone medication use.

Data on tumor characteristics were available for 254 (85.2%) participants from the cancer registry databases and pathology reports, and these women comprised the study group. Information on tumor characteristics was collected at the time of diagnosis, including tumor site, histology, laterality, behavior, grade, stage, tumor size, and ER and progesterone receptor (PgR) status.

#### Cancer registry and SEER linkage

The California Cancer Registry abstracts for all of the California patients were available and were the primary source of tumor characteristic and follow-up data for these patients.

The 60 Connecticut patients were linked with the National Cancer Institute's Surveillance, Epidemiology, and End Results (SEER) Program's 1973–1999 public use data files [12] to obtain tumor characteristics and vital status updates. Because personal identifiers were used only by the centers that conducted the telephone interviews and were not available in the analysis data set for these patients, linkage was performed using the following variables, if these tumor characteristics were also available from pathology reports: year of birth, age at diagnosis, month and year of diagnosis, marital status, race, and tumor characteristics (site, histology, laterality, behavior, grade, stage, ER/PgR status, and size).

There were 60 patients who participated in the Early Onset Breast Cancer Study and who were Connecticut residents at the time of diagnosis. All of the 60 participants were linked to the SEER records using the variables listed above.

#### Pathology reports

Tumor data for the Massachusetts patients were ascertained using pathology reports. Pathology reports were available for 25 of the patients from Massachusetts and were reviewed by a certified tumor registrar and coded for tumor site, histology, laterality, behavior, grade, size, and SEER summary stage. Where available, pathology reports were also used to supplement tumor data for the patients from California and Connecticut.

#### Stage determination

The SEER summary stage classifies tumors into one of the following categories [13]: *in situ*, localized, regional by direct extension, regional by nodes, regional by nodes and direct extension, distant, or unstaged based on the SEER guidelines. Another staging system reported for the patients from California was the TNM staging system, which follows the American Joint Commission on Cancer (AJCC) criteria and consists of assigning appropriate letters or numbers to the following three fields [14]: T (primary tumor), N (nodal involvement), and M (distant metastasis). This classification is recorded for both clinical and pathological staging.

These sources of tumor staging information resulted in a total sample of 247 with stage classification as either localized (invasive carcinoma confined to the breast) or nonlocalized (invasive carcinoma spread beyond the breast, by direct extension and/or to regional lymph nodes, and/or by direct extension beyond adjacent organs specified as regional, metastasis to distant lymph nodes, or development of discontinuous secondary or metastatic tumors).

#### Grade

Tumors were classified according to histologic grade or degree of differentiation, as coded in the registry databases or pathology reports. This measures the degree to which the tumor cells have the differentiated or specialized characteristics of the tissue or organ in which they are found. Tumor grade information was available for 213 participants.

#### Estrogen receptor/progesterone receptor status

Tumors classified as borderline for hormone receptor status were considered positive (*n *= 3 for ER status; *n *= 3 for PgR status). A total of 182 and 176 records contained information on ER and PgR status, respectively.

#### Reproductive/hormonal variables

Reproductive variables and hormonal exposures were assessed using the personal interview data available for all 298 study participants.

#### Family history status

Family history of cancer was assessed during the personal interview. A positive family history was considered to be at least one first-degree female relative (mother or sister) diagnosed with breast or ovarian cancer. Adopted participants were excluded from the family history analyses (*n *= 2), leaving 296 with complete family history information.

### Statistical analysis

#### Tumor presentation

Analyses were completed to assess whether host characteristics (including reproductive/hormonal and family history variables) were associated with tumor characteristics indicative of advanced or aggressive disease (including tumor stage, grade, and ER/PgR status), adjusting for age at diagnosis and education (higher of self or spouse). Odds ratios (ORs) were calculated using unconditional logistic regression with tumor characteristics as the dependent variables (localized versus nonlocalized disease; grade 1–2 tumors versus grade 3–4 tumors; ER positive versus ER negative; PgR positive versus PgR negative). Trend tests for select variables were performed by regressing categorical variables as continuous. All analyses were performed using SAS version 8.2 (SAS Institute Inc., Cary, NC, USA).

#### Survival analysis

Data on follow up were obtained for the California cases through 31 December 2001 from the California Cancer Registry and for the Connecticut cases through 31 December 1999 from the SEER 1999 public use data file [12]. Follow-up data were not available for the patients from Massachusetts. Survival time was truncated at 60 months for the 40 California cases with follow-up information beyond 60 months. A woman's survival time was censored if the woman was known to be alive at the date of last follow up. Kaplan–Meier product limit curves were generated to examine the associations between demographic and reproductive characteristics and overall survival following breast cancer diagnosis. The Wilcoxon test was used to test the equality of the overall survivor functions across groups. The effects of reproductive factors on survival were also quantified with relative risk estimates (and 95% confidence intervals [CIs]) calculated using Cox proportional hazards regression analysis, adjusting for breast cancer stage.

## Results

Demographic and tumor characteristics of the 254 women studied are summarized in Table 1. Seventy-six per cent of tumors were classified as infiltrating duct carcinoma. Stage was localized in 51.6%, nonlocalized in 45.7%, and unknown in 2.8% of cases. The majority of tumors were self-detected (80.7%). Age at diagnosis ranged from 20 to 34 years, with 75.6% of cases occurring between ages 30 and 34 years. The majority of patients (72.8%) were non-Hispanic Caucasians.

### Reproductive and hormonal factors and tumor characteristics

Age- and education-adjusted analyses of reproductive events occurring before diagnosis and tumor characteristics are summarized in Tables 2, 3, 4, 5. Reproductive variables include age at menarche, age at first full-term pregnancy, total number of live births, total number of miscarriages and induced abortions, breastfeeding history, months between last full-term pregnancy and diagnosis, and oral contraceptive use. Tumor characteristics include stage at diagnosis, grade, ER, and PgR status.

Analyses of breast cancer stage included 247 women for whom tumor stage data were available (Table 2). Women with a first full-term pregnancy at age under 20 years were three times as likely to be diagnosed with nonlocalized disease than were women with no full-term pregnancies (OR = 3.0, 95% CI 1.2–7.4). This OR decreased as age at first full-term pregnancy increased (*P *for trend < 0.01). However, when this analysis was restricted to parous women there were no significant associations between age at first full-term pregnancy and stage (for age 20–24 years: OR = 0.7, 95% CI = 0.3–1.7; for age 25–29 years: OR = 0.5, 95% CI = 0.2–1.4; for age 30–34 years: OR = 0.3, 95% CI = 0.1–1.1; versus age <20 years). Women who had three or more live births were about three times more likely to be diagnosed with nonlocalized disease than were women who did not have any live births. When this analysis was restricted to only gravid women, those with three or more pregnancies were more likely to be diagnosed with more advanced stage breast cancer than were women who reported one to two pregnancies (OR = 2.3, 95% CI = 1.2–4.3). Number of births and age at first full-term pregnancy were highly correlated, and when terms for both were included in a logistic regression model neither was found to remain statistically significant (data not shown). Age at menarche, total number of miscarriages, total number of induced abortions, time since last full-term pregnancy, breastfeeding history, and oral contraceptive use were not found to be significantly associated with stage.

Analyses of tumor grade included the 213 women for whom data on tumor grade were available. Early age at first full-term pregnancy was the only reproductive variable examined that was found to be significantly associated with higher tumor grade. Women who had a first full-term pregnancy at age under 20 years were significantly more likely to have tumors of grade 3–4 than were nulliparous women. The other reproductive and family history variables examined were not found to be significantly associated with tumor grade (Table 3).

The analyses of ER and PgR status included the 182 and 176 women, respectively, for whom data on ER and PgR status were available (Tables 4 and 5). Breastfeeding history was the only reproductive variable found to be significantly associated with both ER and PgR status. Among parous women, those who reported ever breastfeeding were significantly more likely to have ER-positive tumors and PgR-positive tumors. Other reproductive variables examined were not found to be significantly associated with either ER or PgR status.

### Family history and tumor characteristics

A family history of breast or ovarian cancer in a mother or sister was examined in relation to tumor characteristics. Having a mother or sister with a diagnosis of breast or ovarian cancer was not significantly associated with tumor stage, grade, ER or PgR status.

### Survival analysis

Follow-up data on vital status and date of last follow up were available for 221 cases. Median 5-year survival time was 45 months (range 2–59 months) for the cases from Connecticut and 54 months (range 3–60 months) for the cases from California, and 52 months overall. Six of the 60 (10.0%) cases from Connecticut and 24 of the 161 cases from California (14.9%) died during the follow-up period.

**Figure 1:**
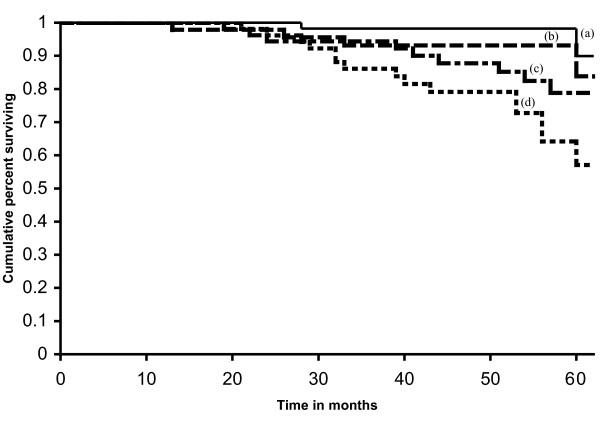
Survival by number of full-term pregnancies and stage. **(a) **Localized tumors, 0–1 full-term pregnancies; **(b) **localized tumors, ≥ 2 full-term pregnancies; **(c) **nonlocalized tumors, 0–1 full-term pregnancies; and **(d) **non-localized tumors, ≥2 full-term pregnancies (*P *= 0.19 for localized tumors; *P *= 0.15 for nonlocalized tumors).

**Figure 2:**
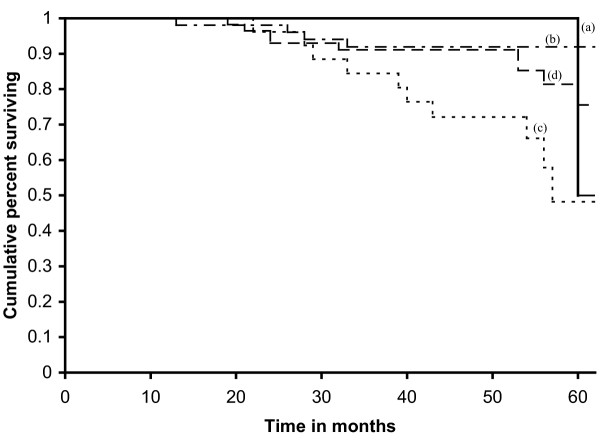
Survival by time since last pregnancy and stage. **(a) **Localized tumors, time since last pregnancy <24 months; **(b) **localized tumors, time since last pregnancy ≥ 24 months; **(c) **nonlocalized tumors, time since last pregnancy <24 months; and **(d) **nonlocalized tumors, time since last pregnancy ≥ 24 months (*P *= 0.24 for localized tumors; *P *= 0.06 for nonlocalized tumors).

Reproductive variables examined in relation to survival were stratified by stage and included number of full-term pregnancies (0–1, ≥ 2), age at first full-term pregnancy (<25 years, 25–34 years, no full-term pregnancies), time since last pregnancy (gravid women: 0–23 months, ≥ 24 months), and lactation history (parous women: never, ever). When stratified by stage, women with either localized or nonlocalized disease who had two or more full-term pregnancies did not have significantly decreased survival as compared with women with no or one full-term pregnancies (*P *= 0.19 and *P *= 0.15, respectively; Fig. 1). However, in an unstratified analysis women with two or more full-term pregnancies had significantly decreased survival as compared with women with no or one full-term pregnancies (*P *< 0.05). Age at first full-term pregnancy and lactation history were not found to be significantly associated with overall survival (*P *= 0.08 and *P *= 0.45, respectively). However, gravid women with nonlocalized tumors who had their last pregnancy within 2 years of diagnosis had borderline decreased survival as compared with gravid women with nonlocalized tumors who had their last pregnancy more than 2 years before diagnosis (*P *= 0.06), but this association was not significant among women with localized tumors (*P *= 0.24; Fig. 2).

Cox proportional hazards regression was used to test the association of reproductive variables (total full-term pregnancies, age at first full-term pregnancy, and time since last pregnancy) with survival, adjusting for stage. In these separate analyses, increased parity ≥ 2 full-term pregnancies as compared with 0–1 full-term pregnancies) was a significant predictor of decreased survival (hazard ratio [HR] = 2.1, 95% CI = 1.0–4.5). Age at first full-term pregnancy and time since last pregnancy were not found to be significantly associated with risk for decreased survival (HR = 0.8, 95% CI = 0.3–1.9, and HR = 0.5, 95% CI = 0.2–1.1, respectively).

## Discussion

Steroid hormones play an important role in the etiology of breast cancer. Evidence has consistently linked endogenous hormone exposure to breast cancer risk [15]. Estrogen has been implicated to induce tumor promotion and/or progression by enhancing cell proliferation [16]. In the present study it was hypothesized that events associated with increases in estrogen levels in young women may influence the progression of breast tumors, resulting in a more rapidly developing disease that presents at a later stage and ultimately has a poorer prognosis. Breast cancer patients younger than 35 years were selected for the study because tumors in this group tend to have the most aggressive profiles.

### Factors associated with tumor characteristics

#### Parity

Assessment of reproductive histories of early-onset breast cancer patients indicated that increased parity was associated with diagnosis of nonlocalized disease. However, this association did not extend to pregnancies ending in either spontaneous or induced abortion. This finding is consistent with a recent study of breast cancer patients aged under 45 years [17] that reported an association between increased parity and overall mortality.

#### Recent pregnancy

Having a full-term pregnancy within the 2 years preceding diagnosis was not found to be associated with later stage disease, higher grade, or ER or PgR status. These findings differ from a recent study of breast cancer patients aged under 45 years [17], which found a recent full-term pregnancy (<2 years before diagnosis) to be associated with disease that had spread to the regional lymph nodes. It should be noted that the present study included a younger population, which may represent a group more likely to have a delay in diagnosis as compared with older premenopausal women.

#### Age at first full-term pregnancy

Another finding of the present study was that early age at first full-term pregnancy was also associated with later stage disease compared with nulliparity. As the age at first full-term pregnancy increased, the risk for having a diagnosis of nonlocalized disease decreased, although risk estimates for any age at first full-term pregnancy were elevated as compared with nulliparity. However, when this analysis was restricted to include only parous women, no significant association between age at first full-term pregnancy and stage was apparent.

There is some previous evidence [18,19] that an early age at first full-term pregnancy may be associated with poorer prognosis. It should also be noted that an early age at first full-term pregnancy has been demonstrated to be protective in terms of lifetime risk for breast cancer [20], and this protective effect may be explained by the process of differentiation of breast tissue induced by a full-term pregnancy, resulting in lower susceptibility to carcinogenic influences. However, the present study addresses the issue of tumor progression rather than the risk for developing breast cancer, because only breast cancer cases were included in these analyses. Therefore, events that are believed to be protective in terms of breast cancer risk, such as early age at first full-term pregnancy and increased parity, may have an adverse effect on breast cancer progression for women who are diagnosed with the disease. Perhaps the timing of reproductive events in relation to the initiation and promotion of breast cancers is critical in determining whether the effect may be harmful for women who develop breast cancer during their principal reproductive years. Several findings of the present study support such a theory.

#### Lactation

Lactation is believed to be associated with decreased circulating levels of estrogen and progesterone, and was hypothesized to be associated with earlier stage tumors, with less aggressive profiles. Lactation among parous women was not found to be associated with tumor stage. However, parous women who reported that they had ever breastfed a child were more likely to have ER-positive and PgR-positive tumors than were parous women who had never breastfed a child. Lactation, therefore, was associated with tumor markers indicative of better prognosis. Another case–control study of women aged 20–74 years [21] found that women who had ever breastfed were at decreased risk for developing ER-positive/PgR-negative (OR = 0.5, 95% CI = 0.3–0.8) and ER-negative/PgR-positive tumors (OR = 0.4, 05% CI = 0.2–0.8). In a case-only analysis, lactation was not associated with ER-positive/PgR-positive or ER-negative/PgR-negative status.

Perhaps the difference in results between the present study and the previous one [21] is in part attributable to the different subpopulations of breast cancer patients. Both ER and PgR status were demonstrated to vary with age, with older women more likely to be diagnosed with tumors positive for these markers [21]. It is possible that, again, the timing of events may be important in determining the effect of lactation on hormone receptor status of breast cancers. In other words, the stage of progression of the breast tumor at the time of lactation may influence whether there is an effect on expression of hormone receptors, and this may differ in general by age of the patient.

#### Exogenous hormones

Exogenous hormone exposure, in the form of oral contraceptives, was also examined in relation to tumor characteristics. This source of exogenous hormones was not associated with tumor stage, grade, or ER or PgR status. Oral contraceptive use was further examined by months of use, but no significant associations between months of use and any of the tumor characteristics emerged. Therefore, the present study does not support an association between exogenous hormone use and tumor presentation among early-onset breast cancer patients.

### Survival analysis

Survival analyses stratified by stage suggested that a recent pregnancy (within 2 years of diagnosis) was associated with decreased survival as compared with women who had not had a recent pregnancy among women with nonlocalized tumors. Thirty-eight per cent of the women with nonlocalized tumors who had a recent pregnancy died within 5 years of diagnosis as compared with 15% of the women who had not had a recent pregnancy. This finding corresponds with previous research reporting that a recent pregnancy has an adverse effect on survival following breast cancer diagnosis [9,10,17,22,23] and supports the idea that a recent pregnancy may be responsible for more rapid progression of disease, resulting in poorer prognosis. The association was not observed among women with localized tumors, perhaps because the survival rate in these women is generally high.

### Strengths and limitations

The hypotheses and findings presented here have biologic plausibility because of the established relationship between estrogen levels and both breast tissue proliferation and breast cancer risk. Estrogen and other hormone exposures are known to be affected substantially by pregnancy and other reproductive events. Therefore, it is biologically plausible that reproductive events may influence the progression of breast cancer among women in their childbearing years via a hormonal pathway.

Including only living cases at the time of the study might have introduced a survival bias among participants. This might have reduced the proportion of women in the study with advanced stage disease. Participants were interviewed within 2 years after diagnosis and, given that breast cancer is generally not a rapidly fatal disease, this may not have introduced much bias. In fact, fewer than 6% of potential participants died before study contact overall.

Another limitation is that there was not much information available regarding screening practices. Although we do not expect women in this age group to be receiving screening mammography, it was not possible to adjust for the effect of screening behavior (breast self examinations or clinical breast examinations) in the analyses. Therefore, if some of the independent variables such as parity and age at first full-term pregnancy are associated with screening practices, then the effects of these independent variables may have been misconstrued. However, it is likely that reproductive events such as pregnancy and childbirth would be associated with more frequent screening procedures because these events place women in the health care system. If so, then the independent effects of these variables on tumor characteristics may have been underestimated.

It is possible that socioeconomic status (SES) confounded the association between reproductive variables and tumor characteristics. Study participants were not well characterized with respect to SES. Education as a surrogate measure of SES was included as a covariate in the analysis, although it was not found to be significantly associated with any of the tumor characteristics under study (stage, grade, or ER or PgR status). Future studies should take care in characterizing participants with respect to SES.

Adjuvant therapy information was not available for all participants and was not included in the survival analyses. It should also be noted that a fairly small sample size limited the extent of multivariate analyses that could be performed. Furthermore, follow-up information was not available for the patients diagnosed in Massachusetts, which further limited the sample size available for survival analyses. However, because breast cancer diagnosis at age under 35 years is a rare event, large sample sizes for population-based studies of this subgroup are often not available, and this study made use of a resource of such patients.

## Conclusion

Reproductive events such as pregnancy play an important role in the etiology of breast cancer. Whether the effect of pregnancy on breast cancer initiation or progression is protective or harmful may depend on the timing of the event in relation to the carcinogenesis process. Women who have had full-term pregnancies, particularly beginning at an early age, have consistently been observed to be at decreased lifetime risk for developing breast cancer as compared with nulliparous women or those who have a first full-term pregnancy at an older age. This may be due in part to the differentiation of breast tissue that occurs during a full-term pregnancy.

The findings of the present study suggest that for women diagnosed with breast cancer before age 35 years, increased parity and an early age at first full-term pregnancy are associated with more advanced stage at presentation. Because all of the women in this study were breast cancer patients, it is possible that the pregnancies had an adverse effect on progression of a breast cancer that was already developing. However, this study suggests that the pregnancy need not be particularly recent to have the adverse effect. Therefore, if a woman has a full-term pregnancy and has not already had a prior initiation event, then the pregnancy would be expected to leave the breast tissue more differentiated and less susceptible to carcinogenic influences in the long term. However, the findings of the present study suggest that a full-term pregnancy may have an adverse effect on a breast cancer that is developing. Studies documenting a transient increase in breast cancer risk following pregnancy also support this theory [6-8].

An understanding of the role of reproductive and hormonal exposures in the etiology and progression of breast cancer in young women highlights some of the age-related issues in breast cancer research by emphasizing that well established risk factors in older women may not have similar effects in the disease process in younger women, whose diagnoses occur nearer in time to reproductive events such as pregnancy.

## Abbreviations

CI = confidence interval; ER = estrogen receptor; HR = hazard ratio; OR = odds ratio; PgR = progesterone receptor; SEER = Surveillance, Epidemiology, and End Results; SES = socioeconomic status.

## Competing interests

The author(s) declare that they have no competing interests.

## Authors' contributions

JAL performed the statistical analysis under the supervision of AZ and wrote the manuscript under the supervision of HA-C. All authors read and approved the final manuscript.

## Figures and Tables

**Table 1:** Demographic and tumor characteristics of early onset breast cancer patients

Characteristic	Frequency (*n*)	%
Age at diagnosis (years)		
20–24	7	2.7
25–29	55	21.6
30–34	192	75.6
Race/ethnicity		
Non-Hispanic white	185	72.8
African-American	17	6.7
Hispanic	33	13.0
Asian/Pacific islander	5	2.0
Native American	1	0.4
Other	13	5.1
Education		
Less than college degree	143	56.3
College graduate	111	43.7
Histology		
Infiltrating duct carcinoma	193	76.0
Comedocarcinoma	15	5.9
Medullary carcinoma	9	3.5
Lobular carcinoma	8	3.2
Infiltrating duct and lobular carcinoma	8	3.2
Other	21	8.3
Stage		
Localized	131	51.6
Nonlocalized	116	45.7
Unknown	7	2.8
Grade/differentiation		
Grade I/well differentiated	9	3.0
Grade II/moderately differentiated	72	24.2
Grade III/poorly differentiated	124	41.6
Grade IV/undifferentiated	8	2.7
Grade not determined/not stated/NA	85	28.5
Laterality		
Right	132	52.0
Left	122	48.0
Estrogen receptor status		
Positive	92	36.2
Negative	87	34.2
Borderline	3	1.2
Unknown	72	28.3
Progesterone receptor status		
Positive	88	34.6
Negative	85	33.5
Borderline	3	1.2
Unknown	78	30.7
Method of breast cancer detection		
Self examination	205	80.7
Physician examination	19	7.5
Mammography	17	6.7
Other	12	4.7
Missing	1	0.4

**Table 2:** Association of stage of breast cancer diagnosis and reproductive and family history variables

Reproductive and family history characteristics	Localized (*n *= 131)	Nonlocalized (*n *= 116)	OR^a^	95% CI	*P*
Age at menarche (years)^b^					
<12	26 (19.8)	29 (25.4)	1.0 (ref)		
≥ 12	105 (80.2)	85 (74.6)	0.8	0.4–1.4	0.35
Age at first full-term pregnancy (years)					
No full-term pregnancies	48 (36.6)	29 (25.0)	1.0 (ref)		
<20	12 (9.2)	19 (16.4)	3.0	1.2–7.4	0.02
20–24	31 (23.7)	35 (30.2)	2.1	1.0–4.3	0.04
25–29	25 (19.1)	24 (20.7)	1.7	0.8–3.6	0.15
30–34	15 (11.4)	9 (7.8)	1.1	0.4–2.9	0.86
Trend					<0.01
Total live births					
0	48 (36.6)	29 (25.0)	1.0 (ref)		
1–2	71 (54.2)	67 (57.8)	1.7	0.9–3.1	0.07
3 or more	12 (9.2)	20 (17.2)	3.1	1.3–7.7	0.01
Trend					0.01
Total miscarriages (gravid women)					
0	69 (74.2)	67 (72.0)	1.0 (ref)		
1 or more	24 (25.8)	26 (28.0)	1.2	0.6–2.3	0.59
Total induced abortions (gravid women)					
0	65 (69.9)	59 (63.4)	1.0 (ref)		
1 or more	28 (30.1)	34 (36.6)	1.2	0.7–2.3	0.51
Breastfeeding (parous women)					
Never	25 (30.1)	26 (29.9)	1.0 (ref)		
Ever	58 (69.9)	61 (70.1)	1.1	0.5–2.2	0.80
Months between last full-term pregnancy and diagnosis					
No full-term pregnancy	48 (36.6)	29 (25.0)	1.0 (ref)		
0–23	23 (17.6)	26 (22.4)	1.9	0.9–3.9	0.09
24 or more	60 (45.8)	61 (52.6)	1.9	1.0–3.5	0.05
Trend					0.06
Oral contraceptive use					
Never	19 (14.5)	21 (18.1)	1.0 (ref)		
Ever	112 (85.5)	95 (81.9)	0.8	0.4–1.5	0.43
Total months of oral contraceptive use^c^					
None	19 (14.6)	21 (18.9)	1.0 (ref)		
1–35	35 (26.9)	29 (26.1)	0.6	0.3–1.3	0.22
36–83	33 (25.4)	29 (26.1)	0.7	0.3–1.4	0.30
84 or more	43 (33.1)	32 (28.8)	0.6	0.3–1.2	0.17
Trend					0.46
Family history of breast or ovarian cancer^d^					
No family history	109 (83.8)	95 (82.6)	1.0 (ref)		
Mother or sister	21 (16.2)	20 (17.4)	1.2	0.6–2.3	0.67

^a^Analyses adjusted for age at diagnosis and education (higher of self or spouse). ^b^Two records missing age at menarche. ^c^Six records missing total months of oral contraceptive use. ^d^Two records were excluded because proband reported being adopted. CI, confidence interval; OR, odds ratio.

**Table 3:** Association of breast cancer grade by reproductive and family history variables

Reproductive and hormonal	Grade 1–2	Grade 3–4	OR (for grade	95% CI	*P*
Age at menarche (years)					
<12	15 (18.8)	33 (25.4)	1.0 (ref)		
≥ 12	65 (81.2)	97 (74.6)	0.7	0.4–1.5	0.38
Age at first full-term pregnancy (years)					
No full-term pregnancies	27 (33.3)	35 (26.5)	1.0 (ref)		
<20	5 (6.2)	23 (17.4)	3.2	1.0–9.9	0.05
20–24	25 (30.9)	34 (25.8)	1.0	0.4–2.1	0.95
25–29	17 (21.0)	26 (19.7)	1.4	0.6–3.1	0.43
30–34	7 (8.6)	14 (10.6)	2.5	0.8–7.5	0.10
Trend					0.27
Total live births					
0	27 (33.3)	35 (26.5)	1.0 (ref)		
1–2	45 (55.6)	78 (58.3)	1.5	0.8–2.8	0.25
3 or more	9 (11.1)	19 (15.2)	1.7	0.6–4.6	0.30
Trend					0.22
Total miscarriages (gravid women)					
0	50 (82.0)	73 (70.2)	1.0 (ref)		
1 or more	11 (18.0)	31 (29.8)	2.0	0.9–4.5	0.08
Total induced abortions (gravid women)					
0	40 (65.6)	68 (65.4)	1.0 (ref)		
1 or more	21 (34.4)	36 (34.6)	1.0	0.5–1.9	0.92
Breastfeeding (parous women)					
Never	15 (27.8)	34 (35.0)	1.0 (ref)		
Ever	39 (72.2)	63 (65.0)	0.8	0.4–1.6	0.51
Months between last full-term pregnancy and diagnosis					
No full-term pregnancy	27 (33.3)	35 (26.5)	1.0 (ref)		
0–23	19 (23.4)	27 (20.4)	1.2	0.5–2.6	0.71
≥ 24	35 (43.2)	70 (53.0)	1.7	0.9–3.5	0.12
Trend					0.12
Oral contraceptive use					
Never	10 (12.4)	21 (15.9)	1.0 (ref)		
Ever	71 (87.6)	111 (84.1)	0.7	0.3–1.6	0.42
Total months of oral contraceptive use^b^					
None	10 (13.2)	21 (16.0)	1.0 (ref)		
1–35	24 (31.6)	32 (24.4)	0.8	0.4–2.0	0.68
36–83	17 (22.4)	38 (29.0)	1.5	0.6–3.7	0.35
84 or more	25 (32.9)	40 (30.5)	1.2	0.5–2.8	0.64
Trend					0.78
Family history of breast or ovarian cancer^c^					
No family history	65 (80.2)	113 (86.9)	1.0 (ref)		
Mother or sister	16 (19.8)	17 (13.1)	0.7	0.3–1.4	0.29

^a^Analyses adjusted for age at diagnosis and education (higher of self or spouse). ^b^Five records missing total months of oral contraceptive use. ^c^Two records were excluded because proband reported being adopted. CI, confidence interval; OR, odds ratio.

**Table 4:** Association of estrogen receptor status and reproductive and family history variables

Reproductive and family history characteristics	ER-positive or borderline tumors (*n *= 95)	ER-negative tumors (*n *= 87)	OR^a^	95% CI	*P*
Age at menarche (years)^b^					
<12	16 (17.0)	19 (22.4)	1.0 (ref)		
≥ 12	78 (83.0)	66 (77.6)	0.7	0.3–1.5	0.42
Age at first full-term pregnancy (years)					
No full-term pregnancies	27 (28.4)	27 (31.0)	1.0 (ref)		
<20	10 (10.5)	16 (18.4)	1.5	0.6–4.0	0.43
20–24	26 (27.4)	19 (21.8)	0.7	0.3–1.6	0.39
25–29	23 (24.2)	16 (18.4)	0.7	0.3–1.7	0.45
30–34	9 (9.5)	9 (10.3)	1.2	0.4–3.6	0.80
Trend					1.0
Total live births					
0	27 (28.4)	27 (31.0)	1.0 (ref)		
1–2	57 (60.0)	49 (56.3)	0.9	0.4–1.7	0.68
3 or more	11 (11.6)	11 (12.6)	1.0	0.3–2.8	0.97
Trend					0.86
Total miscarriages (gravid women)					
0	61 (80.3)	42 (65.6)	1.0 (ref)		
1 or more	15 (19.7)	22 (34.4)	2.1	1.0–4.6	0.05
Total induced abortions (gravid women)					
0	48 (63.2)	44 (68.8)	1.0 (ref)		
1 or more	28 (26.8)	20 (31.2)	0.7	0.4–1.5	0.42
Breastfeeding (parous women)					
Never	12 (17.6)	29 (48.3)	1.0 (ref)		
Ever	56 (82.4)	31 (51.7)	0.2	0.1–0.5	<0.001
Months between last full-term pregnancy and diagnosis					
No full-term pregnancy	27 (28.4)	27 (31.0)	1.0 (ref)		
0–23	19 (20.0)	16 (18.4)	0.9	0.4–2.0	0.71
≥ 24	49 (51.6)	44 (50.6)	0.9	0.4–1.8	0.77
Trend					0.78
Oral contraceptive use					
Never	19 (20.0)	12 (13.8)	1.0 (ref)		
Ever	76 (80.0)	75 (86.2)	1.6	0.7–3.4	0.28
Total months of oral contraceptive use^c^					
None	19 (20.2)	12 (14.6)	1.0 (ref)		
1–35	24 (25.5)	23 (28.0)	1.1	0.5–2.6	0.81
36–83	16 (17.0)	20 (24.4)	1.5	0.6–3.7	0.42
84 or more	35 (37.2)	27 (32.9)	0.9	0.4–2.1	0.85
Trend					0.78
Family history of breast or ovarian cancer^d^					
No family history	75 (79.8)	71 (82.6)	1.0 (ref)		
Mother or sister	19 (20.2)	15 (17.4)	0.9	0.4–1.9	0.72

^a^Analyses adjusted for age at diagnosis and education (higher of self or spouse).

^b^Three records missing age at menarche. ^c^Six records missing total months of oral contraceptive use. ^d^Two records were excluded because proband reported being adopted. CI, confidence interval; ER, estrogen receptor; OR, odds ratio.

**Table 5:** Association of progesterone receptor status and reproductive and family history variables

Reproductive and family history characteristics	PgR-positive or borderline tumors (*n *= 91)	PgR-negative tumors (*n *= 85)	OR^a^	95% CI	*P*
Age at menarche (years)^b^					
<12	14 (15.6)	21 (25.3)	1.0 (ref)		
≥ 12	76 (84.4)	62 (74.7)	0.6	0.3–1.2	0.14
Age at first full-term pregnancy					
No full-term pregnancies	27 (29.7)	25 (29.4)	1.0 (ref)		
<20	10 (11.0)	15 (17.6)	1.6	0.6–4.4	0.37
20–24	25 (27.5)	20 (23.5)	0.8	0.4–2.0	0.71
25–29	19 (20.9)	18 (21.2)	1.1	0.5–2.6	0.82
30–34	10 (11.0)	7 (8.2)	0.9	0.3–2.9	0.88
Trend					0.65
Total live births					
0	27 (29.7)	25 (29.4)	1.0 (ref)		
1–2	53 (58.2)	50 (58.8)	1.1	0.5–2.1	0.87
3 or more	11 (12.1)	10 (11.8)	1.0	0.3–2.9	1.00
Trend					0.95
Total miscarriages (gravid women)					
0	54 (76.1)	45 (69.2)	1.0 (ref)		
1 or more	17 (23.9)	20 (30.8)	1.4	0.7–3.0	0.38
Total induced abortions (gravid women)					
0	46 (64.8)	44 (67.7)	1.0 (ref)		
1 or more	25 (35.2)	21 (32.3)	0.8	0.4–1.7	0.65
Breastfeeding (parous women)					
Never	14 (21.9)	25 (41.7)	1.0 (ref)		
Ever	50 (78.1)	35 (58.3)	0.4	0.2–0.8	0.02
Months between last full-term pregnancy and diagnosis					
No full-term pregnancy	27 (29.7)	25 (29.4)	1.0 (ref)		
0–23	17 (18.7)	16 (18.8)	1.0	0.4–2.5	0.95
≥ 24	47 (51.6)	44 (51.8)	1.1	0.5–2.2	0.87
Trend					0.87
Oral contraceptive use					
Never	19 (20.9)	10 (11.8)	1.0 (ref)		
Ever	72 (79.1)	75 (88.2)	2.0	0.8–4.6	0.11
Total months of oral contraceptive use^c^					
None	19 (21.3)	10 (12.2)	1.0 (ref)		
1–35	23 (25.8)	23 (28.0)	1.6	0.6–3.9	0.33
36–83	14 (15.7)	20 (24.4)	2.3	0.9–6.1	0.10
84 or more	33 (37.1)	29 (35.4)	1.5	0.6–3.5	0.38
Trend					0.34
Family history of breast or ovarian cancer^d^					
No family history	71 (78.9)	70 (83.3)	1.0 (ref)		
Mother or sister	19 (21.1)	14 (16.7)	0.8	0.4–1.8	0.58

^a^Analyses adjusted for age at diagnosis and education (higher of self or spouse).

^b^Three records missing age at menarche. ^c^Five records missing total months of oral contraceptive use. ^d^Two records were excluded because proband reported being adopted. CI, confidence interval; PgR, progesterone receptor; OR, odds ratio.
